# Microphysical space of a liver sinusoid device enables simplified long-term maintenance of chimeric mouse-expanded human hepatocytes

**DOI:** 10.1007/s10544-014-9877-x

**Published:** 2014-06-07

**Authors:** Steven P. Maher, Richard B. Crouse, Amy J. Conway, Emilee C. Bannister, Anil Kumar H. Achyuta, Amy Y. Clark, Francy L. Sinatra, Joseph D. Cuiffi, John H. Adams, Dennis E. Kyle, Wajeeh M. Saadi

**Affiliations:** 1 Bioengineering Center at USF, Charles Stark Draper Laboratory, 3802 Spectrum Blvd ste 201, Tampa, FL 33612 USA; 2 Department of Global Health, University of South Florida, 3720 Spectrum Blvd ste 304, Tampa, FL 33612 USA

**Keywords:** Organ microenvironment, Microfluidics, Human hepatocytes, Factor IX, CYP3A4, Albumin

## Abstract

While many advanced liver models support hepatic phenotypes necessary for drug and disease studies, these models are characterized by intricate features such as co-culture with one of more supporting cell types or advanced media perfusion systems. These systems have helped elucidate some of the critical biophysical features missing from standard well-plate based hepatocyte culture, but their advanced designs add to their complexity. Additionally, regardless of the culture system, primary hepatocyte culture systems suffer from reproducibility issues due to phenotypic variation and expensive, limited supplies of donor lots. Here we describe a microfluidic bilayer device that sustains primary human hepatocyte phenotypes, including albumin production, factor IX production, cytochrome P450 3A4 drug metabolism and bile canaliculi formation for at least 14 days in a simple monoculture format with static media. Using a variety of channel architectures, we describe how primary cell phenotype is promoted by spatial confinement within the microfluidic channel, without the need for perfusion or co-culture. By sourcing human hepatocytes expanded in the Fah, Rag2, and Il2rg-knockout (FRG™-KO) humanized mouse model, utilizing a few hundred hepatocytes within each channel, and maintaining hepatocyte function for weeks *in vitro* within a relatively simple model, we demonstrate a basic primary human hepatocyte culture system that addresses many of the major hurdles in human hepatocyte culture research.

**Electronic supplementary material:**

The online version of this article (doi:10.1007/s10544-014-9877-x) contains supplementary material, which is available to authorized users.

## Introduction

The liver is involved in the course of many infectious and non-infectious diseases as it maintains hundreds of biological functions and is the primary organ responsible for activation and clearance of most therapeutic drugs. While some studies, such as hepatic drug metabolism and hepatotoxicity screening, can utilize short-term hepatocyte cultures or liver microsomes, long-term culture technologies are needed to model most liver diseases and their potential therapies (Donato et al. 2008). Previous liver culture studies with the causative agents of viral hepatitis and malaria have demonstrated that such models require at least three weeks of continuous host cell culture (Mazier et al. 1984, Ploss et al. 2010, March et al. 2013). Also, despite their limited availablity and lot-to-lot variation, these and other studies have found human hepatocytes essential in order to properly model both the disease and the human response to potential therapeutics. Thus, a complete infectious disease liver model for drug discovery should incorporate a renewable source of human hepatocytes in a simple, long term, and flexible culture system.

Many seeding techniques, culture methods, and microfluidic culture systems have been developed to accomplish long-term hepatocyte culture (reviewed in Soldatow et al. 2013). Media perfusion has been shown to be central for maintenance of sufficient oxygen transport and appropriate cytokine gradients, and results in enhanced phenotypic gene expression from hepatic cell lines (Domansky et al. 2010; Prot et al. 2011). Primary hepatocyte vesicular transport functions require cuboidal cell morphology with proper apical and basolateral surface domains; these domains are established, in part, by proper interaction with extracellular matrix proteins. Culturing hepatocytes with specific extracellular matrix compositions significantly affects hepatic phenotypes, and an overlay of extracellular matrix can help maintain cuboidal morphology and hepatocyte polarization (Flaim et al. 2005, LeCluyse 2001). Cuboidal cell architecture is also affected by the culture microenvironment; compaction within a small channel leads to extended viability (Lee et al. 2007). To enable multiplexing, maintenance of primary hepatocyte phenotype in a multiwell format has been achieved and utilized by patterning hepatocytes in a co-culture with fibroblasts, demonstrating the importance of cell-cell interaction in the design of high-order culture systems (Bhatia et al. 1997; Khetani and Bhatia 2008).

While these elements are critical for the development of a high-order liver model, they also carry drawbacks. Perfusion often requires integrated pumps and custom-fabricated fluidic lines. Cells cultured on single component extracellular matrices show matrix lot-specific phenotypes and multicomponent matrices are often difficult to mix and deposit on surfaces; requiring additional technologies. Co-cultures require specific, often proprietary sub-clones of supporting cells and require much trial and error to optimize seeding logistics and media compositions. These characteristics make it difficult to incorporate several functionalities into one culture system to take advantage of the additive effects of each mechanism.

To address the challenges of integrating multiple mechanisms into one system for long-term, stable culture of human hepatocytes, we have designed a microfluidic bilayer device (MBD) featuring two microfluidic channels separated by a polydimethylsiloxane (PDMS) membrane. While this MBD is capable of media perfusion, zonal deposition of extracellular matrix, and cell co-culture, we describe how static culture of hepatocytes in the MDB is sufficient to maintain hepatocyte phenotype for three weeks, without the need for perfusion or co-culture. We demonstrate that this phenotype is dependent on spatial confinement of hepatocytes into a collagen and fibronectin-coated microphysical space, which is relevant to a liver sinusoid. To enable comparisons between studies, to model the human response to novel compounds, and to take advantage of their potentially unlimited production, we selected commercially available FRG™-KO mouse-expanded human hepatocytes (FHH’s) as host cells (Azuma et al. 2007, Strom et al. 2010). This new technology shows promise for mitigation of risks associated with primary human hepatocyte lots, particularly their limited availability and reproducibility. Here we describe an optically accessible and simple, yet highly adaptable and reproducible liver model. This model provides a testbed to measure the incremental advantages of adding important factors such as media perfusion, complex extracellular matrices, and co-cultures to create an even more effective liver model. Our overarching goal is to develop and optimize a platform that recapitulates key hepatocyte phenotypes for long-term studies of multiple liver-associated diseases, such as hepatitis and malaria, and enables testing of potential therapies.

## Materials and methods

### Fabrication

**Fig. 1:**
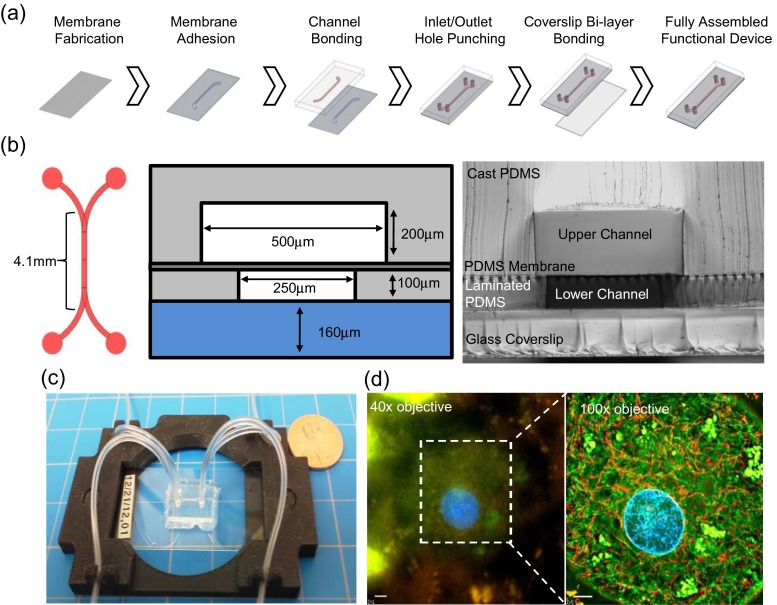
MBD fabrication and features. (**a**) A Spun PDMS membrane is bonded to a cast PDMS upper channel and a laminated PDMS lower channel before being bonded to a glass coverslip. (**b**) Schematic top-view of channel layout (left); cross-sectional schematic (middle) and microscope image (right) showing device dimensions and arrangement of the different layers. Scale bar represents 100 μm. (**c**) Complete device is seated into a 3D-printed microscope stage adapter. (**d**) Device dimensions are thin enough for high resolution, individual tubulin strands and mitochondrion within FHH’s are noticeable by imaging with a 100x objective but not a 40x objective. Green: tubulin, red: mitochondria, blue: DNA. Scale bars represent 5 μm

In summary, the MBD consists of a No. 1 glass coverslip bonded to a 100 μm-thick slab of PDMS featuring a 250-μm-wide, 7.4-mm-long laminated channel. A 10-μm-thick spun PDMS membrane, with a 7.25 % total porosity of evenly-patterned 10-μm-diameter holes, separates the lower and upper layers, thus forming bilayer channels. The upper layer consists of a 500-μm-thick PDMS slab featuring a 500-μm-wide, 7.4-mm-long cast channel. The two channels originate in separate areas and run together for 4.1 mm, forming the bilayer (Fig. 1).

The MBD is assembled by first fabricating the membrane, upper channel, and lower channel out of PDMS, followed by plasma bonding of the layers to each other and then to a glass coverslip. The membrane is fabricated by mixing PDMS base and curing agent at 10:1 w/w (PDMS, Sylgard® 184, Dow Corning, Midland, MI, U.S.A.), then degassing the mixture for 30 min before pouring approximately 1 g onto an SU8 mold featuring 10-μm-tall, evenly spaced posts. The wafer (treated after initial lithography with (tridecafluoro- 1,1,2,2-tetrahydroctyl) trichlorosilane (Gelest, Morrisville, PA) for 2 days) is then spun at 500 rpm for 60 s, then 6000 rpm for 250 s (10 s ramp). The spun wafer is placed on a 100 °C hot plate for 10 min, covered in Kapton® and soaked in IPA before the PDMS membrane is cut and pulled off the wafer. The lower layer is fabricated from modified protocols previously described (Epshteyn et al. 2011). Briefly, the lower, laminated layer is manufactured by pouring 1.2–1.5 g of degassed PDMS (mixed as above) onto an SU8 wafer with inverse channel features. The wafer is placed in a laminator with Kapton above the PDMS, needed for handling the thin and fragile laminated layer after pressurizing at 35 psi, 60 °C for 30 min. The upper layer is fabricated by seating an SU8 wafer with inverse channel features into a petri dish and then pouring degassed PDMS (mixed as above) onto the wafer to a height of 2 mm and allowing it to cure at 65 °C for 90 min. Once cured, the upper layer containing the cast channel feature is cut away and inlet/outlet holes are punched with a 1 mm biopsy punch (Fig. 1).

Final assembly of the microfluidic MBD begins by activating the lower laminated layer and membrane in a plasma asher under the following conditions: time: 10 s, RF power: 300 W, vacuum set point: 0.180 torr. The lower laminated layer and membrane are then pressed together under a weight at 65 °C for 20 min. The inlet/outlet holes for these layers are punched with a 1 mm biopsy punch. Then, the upper cast layer and reverse side of the membrane-lower layer are activated in a plasma asher (conditions above), channels and inlet/outlet holes are aligned, and the layers are pressed together under a weight at 65 °C for 20 min. Lastly, the PDMS layer stack and a No. 1 glass coverslip (Dow Corning, Midland, MI, U.S.A.) are activated in a plasma asher (conditions above) and pressed together. A thin layer of silicon glue is applied to the outside interface between PDMS and glass in order to improve MBD ruggedness during handling. Completed MBD’s are ethylene oxide sterilized for 12 h.

### Cell seeding and culture

Stocks of human liver cell line HC-04 (Sattabongkot et al. 2006) were maintained in media consisting of a 1:1 mixture of F12 and MEM alpha (Life Technologies™, Grand Island, NY, U.S.A.) supplemented with 0.03 mM Hepes, 551 mg/L l-Glutamine, and 10 % FBS (Thermo Scientific® Hyclone®, Waltham, MA, U.S.A.). FHH’s (Yecuris™, Tualatin, OR, U.S.A.) of the same human donor but from two lots (two engraftments and harvestings from FRG™-KO mice), were maintained in Hepatocyte Culture Medium supplemented with a SingleQuot™ kit (Lonza, Walkersville, MD, U.S.A.) containing ascorbic acid, fatty acid free BSA, transferrin, insulin, hEGF and hydrocortisone, prepared per manufacturer’s instructions. Human Liver Sinusoidal Endothelial Cells (HLSEC’s, Sciencell™, Carlsbad, CA, U.S.A.) were thawed per manufacturer’s instructions onto a 2 μg/cm^2^ fibronectin-coated flask (Sigma-Aldrich®, St. Loius, MO, U.S.A.) and cultured in manufacturer’s complete medium. HLSEC’s were allowed grow to 90 % confluence in the flask before being shaken in an orbital shaker at 1 000 rpm overnight, to align the cells and adapt them to shear stress (Fig. S5). All cells were maintained in a 5 % CO2 incubator at 37 °C.

The net volume of the upper and lower channels of the MBD is less than 4 μL and cannot be directly accessed by pipette tips. To enable liquid handling, 0.02 in ID, 0.06 in OD Tygon® Tubing (US Plastics®, Lima, Ohio, U.S.A.) is inserted into the slightly smaller diameter inlets and outlets, creating a tight but temporary seal. A 50 μL HPLC syringe with a 22 gauge blunt needle is used to perfuse extracellular matrix solution, media, and cells into the MBD by inserting the blunt tip into the opposite end of the Tygon tubing. For static culture, the tubing is removed from a seeded MBD and the lower 1 cm of a 1 mL pipette tip (Eppendorf, Hauppauge, NY, U.S.A.) is cut, sterilized, and inserted into the MBD to serve as a media reservoir. Four tips, one inserted into each of the four inlets/outlets, hold about 120 μL of media. Alternatively, for perfused culture, after cell seeding an extended piece of Tygon tubing is connect to a syringe by a 22 gauge blunt needle tip. The syringe sits in a syringe pump (Harvard Apparatus, Holliston, MA, U.S.A.) and a flow rate of 137 μL/h is equivalent to a shear stress of 0.1 dyn/cm^2^ at the cell surface is applied in the lower channel (Fig S3).

The MBD can support long-term culture of either cell lines, such as HepG2 and HC-04, or FHH’s. Additionally, the MBD supports both direct and indirect co-cultures when cells are seeded together in one channel or separately in either channel (Fig. 5). Because fibronectin-coated surfaces are recommended for endothelial cells and collagen-coated surfaces are recommended for hepatocytes, MBD’s were coated at a calculated concentration of 5 μg/cm^2^ collagen and 2 μg/cm^2^ fibronectin by injecting a solution 1.4 μg/μL rat tail collagen I (BD™, Waltham, MA, U.S.A.) and 0.6 μg/μL bovine fibronectin (Sigma) diluted in 0.01 N acetic acid into the upper and lower channels, allowing it to adsorb onto the surface overnight. For hepatocyte seeding, a vial of cryopreserved human FHH’s was thawed according to manufacturer’s protocols; for hepatocyte cell lines, cells are trypsin-released from a flask, pelleted, and resuspended in complete hepatocyte culture media to a concentration of 15 000 cells/μL (for cell lines) or 8 000 cells/μL (for FHH’s). The cell slurry is perfused into the MBD lower channel with a 50 μL syringe and allowed to adhere over 3 h before initiation of static culture, described above. For HLSEC-FHH co-culture experiments, hepatocyte seeding was preceded by trypsin-releasing aligned HLSEC’s, concentrating them to 2 400 cells/μL, injecting them into the upper channel, and allowing them to grow to confluence over 2 days under static media. During static MBD culture, media is changed daily with the appropriate hepatocyte culture medium (for hepatocyte-only cultures) or a 1:1 mixture of hepatocyte culture media and endothelial culture media (for HLSEC and hepatocyte co-cultures). FHH’s were seeded at a concentration of 100,000 cells/well into 8-chamber slides (BD) coated with 5 μg/cm^2^ rat tail collagen I and 2 μg/cm^2^ fibronectin, serving as control cultures for albumin production, factor IX and bile canaliculi experiments.

### Imaging, sampling, and phenotyping sssays

For high-resolution imaging of FHH’s within the MBD, hepatocytes cultured for 12 days within the MBD were stained with 10 μg/mL Hoechst 33 342 (Life Technologies), 10 μM Vybrant Celltracker Red (Life Technologies) and 250 nM Tubulin Tracker Green (Life Technologies) for 30 min. Live, stained cells were imaged with a 40x and 100x objective on a Deltavision Elite (Applied Precision, Issaquah, WA, U.S.A.) and, for 100x images, were deconvoluted using the softWoRx® image processing software (Applied Precision). Assessing bile production was performed by incubating MBD cultures with 2 μg/mL 5-(and-6)-Carboxy-2′,7′-Dichlorofluorescein Diacetate (Carboxy-DCFDA, Life Technologies) and 10 μg/mL Hoechst 33 342 for 10 min, washing the MBD with complete medium, and imaging live cells on a Deltavision Elite (Khetani and Bhatia 2008). HLSEC-hepatocyte co-cultures within the MBD were imaged by first seeding the MBD with HLSEC’s and allowing them to grow to confluence over 2 days before staining with 10 μM Vybrant® Celltracker Red. Simultaneously, HC-04 hepatocytes were stained with 10 μM Vybrant Celltracker Green within a culture flask before being trypsin-released and seeded into the vascularized MBD. After attachment, the MBD was imaged by a Deltavision Elite from coverslip to upper channel at 1 μm intervals to make a Z-stack; which was further modeled into polygons in 3D space by the softWoRx image processing package. Viability staining of cell lines and FHH’s after long term-flow and static culture was performed with the Live/Dead® Calcein AM/Ethidium Homodimer kit (Life Technology) per manufacturer’s protocol.

For albumin and factor IX production assays, media was collected from the pipette tip reservoirs or 8 chamber slide wells during daily media changes and frozen at -80 °C until needed for the ELISA. Samples were loaded into an ELISA specific for human albumin (Bethyl Labs, Montgomery, TX, U.S.A.) or human factor IX (Assaypro, St. Charles, MO, U.S.A.) after diluting 1:10 v/v (for albumin) or 1:5 v/v (for factor IX) in kit-supplied dilution buffer. ELISA labeling and washing was performed per manufacturer’s protocols, and final concentrations were corrected for dilution factor. For CYP3A4 assays, MBD cultures were induced with 25 mM Rifampicin (MP Biomedical™, Santa Ana, CA, U.S.A.) or DMSO for 3 days. The assay was performed with a CYP3A4 P450-Glo™ kit (Promega, Madison, WI, U.S.A.) using a slight modification to manufacturer’s lytic protocol: MBD’s were treated with 100 μL of the luciferin reagent for 45 min and then collected into an opaque plate, followed by 100 μL of cell lysis reagent injected through the MBD before being mixed with the previously collected luciferin reagent, to collect both intracellular and extracellular luciferin from the MBD. Statistics were performed with Prism (Graphpad, La Jolla, CA, U.S.A.) using multiple student’s t-tests.

## Results and discussion

### Device design and optimization

**Fig. 2:**
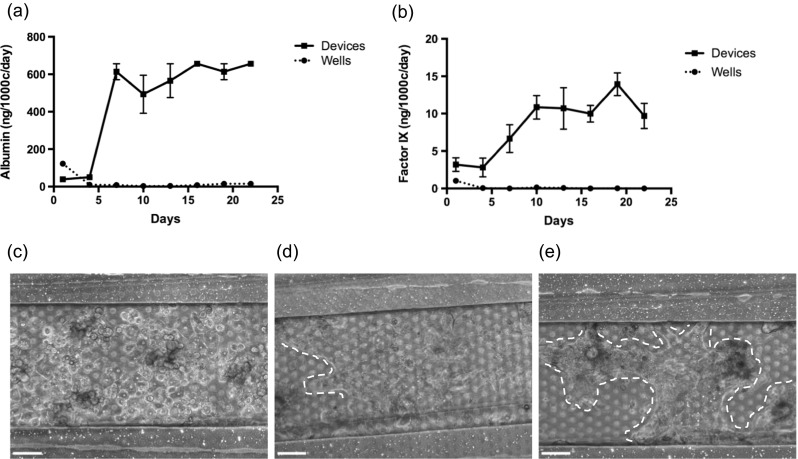
Three-week maintenance of albumin secretion and morphology. (**a**, **b**) FHH wellplate and MBD albumin and factor IX output over three weeks in static culture show a stabilization of primary phenotype in devices but not wells. Error bars represent standard error of the mean (SEM) (n = 4). (**c**, **d**, **e**) MBD-cultured FHH’s imaged at 5 h, 13 days, and 21 days post-seed, respectively, showing that FHH’s become more cuboidal and form multicellular structures, outlined by dotted lines. Scale bars represent 100 μm

Channel materials and dimension were designed around several considerations for optimization. First, functional liver research is hindered by the cost of primary human hepatocytes, regardless of sourcing from human cadavers or further propagation within the FRG™-KO mouse. Thus, we designed the MBD to hold only 200 hepatocytes per sample, versus 10,000 or more for conventional well plate-based culture, such that a single cryovial of hepatocytes could populate dozens of devices at once. Second, the device was optimized to allow static culture without perfusion, thus simplifying device implementation by eliminating the need for syringe pumps. To minimize diffusion limitations, we shortened the MBD channel length, compared to a precursor channel described in Epshteyn et al. (2011), such that no part of the channel was more than 5 mm from an inlet. Cells cultured beyond a threshold of 5 mm from an inlet die; presumably due to lack of media exchange by diffusion alone during static culture (Fig. S2). Third, the MBD is meant to represent the functional unit of the liver. At the cellular level, the liver is organized into single or double layered sheets of hepatocytes separated by endothelial cell-lined sinusoids. The lower channel of the device is approximately 100 μm tall, 250 μm wide and 4 mm long in the overlapping area. Upon seeding, a monolayer of primary hepatocytes about four to five cells wide occupies the length of the channel across from the vascular upper channel, similar to the liver sinusoid (Fig. 2). Lastly, long-term culture within a complex, non-well platform is challenging because the various materials and interfaces used to manufacture the device are exposed to water for extended timelines; increasing the opportunity for delamination. To facilitate lamination by plasma bonding, which results in much stronger bonds than those obtained with either glue or silane treatment, a PDMS membrane was developed to yield an entirely glass and PDMS device (Fig. 1). This membrane was fabricated using a well-known soft-lithographic molding process (Jackman et al. 1999); surface treatment and spin-speeds were optimized to ensure a reproducible fabrication process with >90 % yield. Successful fabrication of open holes was verified with SEM (Fig. S1), and the number of open holes estimated to be ~98 %. PDMS is useful as a prototyping substrate but is not recommended for studies involving small molecules because PDMS is known to absorb hydrophobic molecules; a potential confounder in drug dosing experiments (Regehr et al. 2009). This model is a prototype and as the exact architectures and cell types needed for a long-term liver model are better understood, a future device composed of non-PDMS plastics and manufactured via rapid prototyping or injection molding would circumvent the negative properties of a PDMS-based device.

### Long-term maintenance of primary human hepatocyte phenotypes

**Fig. 3:**
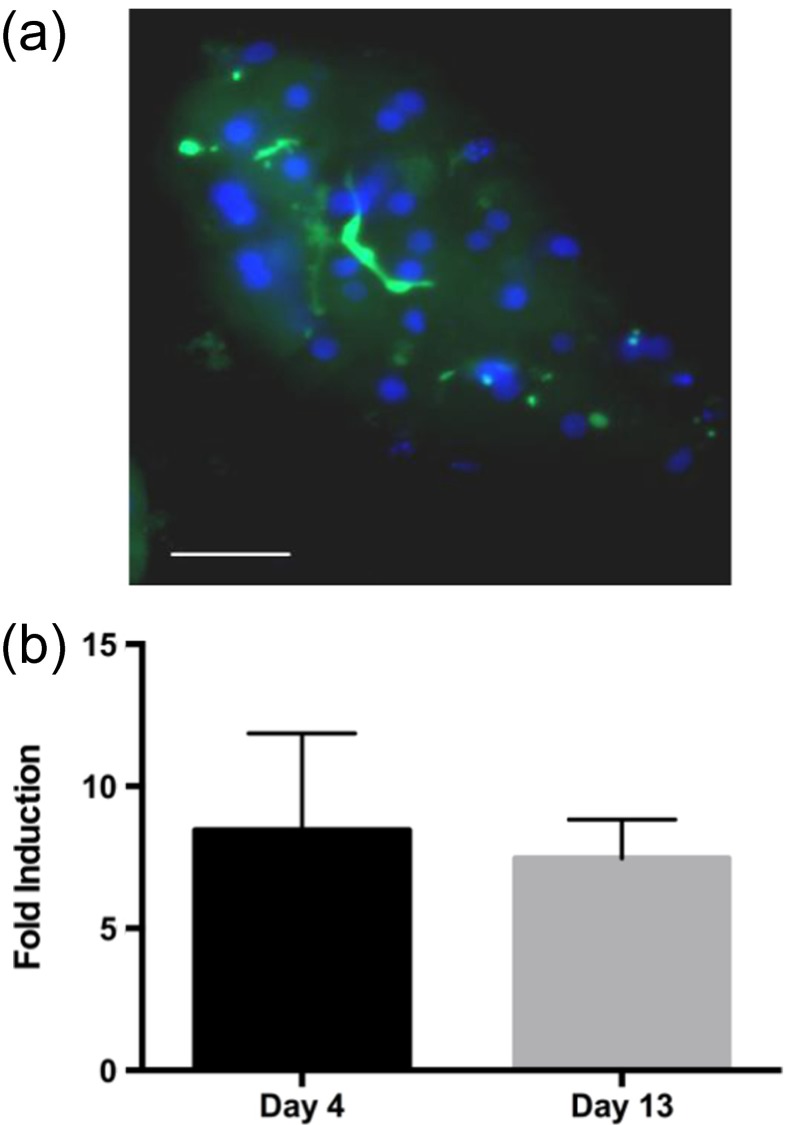
Hepatocyte P450 expression and bile canaliculi formation. (**a**) At two weeks post seed of FHH’s in the MBD, bile production identified by Carboxy-DCFDA dye, which fluoresces upon cleavage by intercellular esterases and is subsequently pumped into extracellular bile networks, green: CDF, blue: DNA. (**b**) Rifampin-induced CYP3A4 expression is maintained for at least two weeks in MBD culture. Error bars represent SEM (n = 2). Scale bar represents 50 μm

Four of the many fundamental roles of hepatocytes are the production of serum albumin and clotting factor IX, production and excretion of bile salts, and activation of the cytochrome P450 enzymes. These phenotypes are lost within a few days of typical *in vitro* well-plate culture (Guguen-Guillouzo et al. 1983; Miao et al. 2000). On the contrary, primary hepatocytes can maintain their phenotype for ≥ 3 weeks in the MBD in static culture (no perfusion, with daily refreshing of the media), without any other supporting cells in the device. Upon seeding in the lower MBD channel, FHH’s adhere to the glass coverslip in a monolayer. Over the course of several days hepatocyte groups ranging from five to twelve or more cells remodel into multicellular structures, becoming more cuboidal as observed by microscopy (Figs. 2 & 3). This remodeling phenomenon appears essential for function, as it is accompanied with a simultaneous rise in albumin and factor IX production during the first ten days post seed, which then stabilizes (Fig. 2). This production level remained stable until we stopped the experiment 21 days post-seed. Formation of extracellular bile canaliculi progressed in tandem with the formation of cuboidal multicellular structures over ten or more days within the MBD. Bile canaliculi are representative of hepatocytes maintaining proper polarization, with two basolateral surfaces separated by an apical belt featuring transporters for bile salts into canaliculi between neighboring hepatocytes (Dunn et al. 1991). FHH’s cultured in standard wells spread and do not produce bile canaliculi (data not shown, Nakao et al. 2011) but FHH’s cultured within the MBD formed bile networks visualized by Carboxy-DCFDA; a dye which fluoresces upon cleavage by hepatic esterases and is subsequently pumped into bile networks (Fig. 3)

A valid *in vitro* liver model, especially one designed to model both the disease and potential treatments, must demonstrate sustained cytochrome P450 activity to project drug activation and clearance rates. Quantitative gene expression measured by RT-PCR is often used to measure many of the P450 induction levels as many of the dozens of P450 enzymes can be quantified simultaneously, but this method does not reveal actual metabolism (Rodríguez-Antona et al. 2001). Therefore, metabolism of FHH’s within MBD’s was measured by cleavage of luciferin from a CYP3A4 substrate after induction with Rifampin over three days. While in standard well cultures metabolism rapidly drops and becomes nonexistent within days of hepatocyte seeding, expression and function of CYP3A4 was maintained for at least two weeks in MBD culture (Fig. 3). While more studies are needed to validate other P450 enzymes, CYP3A4 is responsible for metabolism of over 30 % of FDA approved drugs and is representative of *in vitro* hepatic drug metabolism (Guengerich 1999).

Many alternative culture methods utilizing co-cultures, gel overlays, and perfused channels have demonstrated these phenomena. However, only the MBD promotes hepatocyte culture with key hepatocyte phenotypes without co-culture or perfusion. While unexpected, this result is consistent with studies showing that hepatocytes seeded within tubes or cords demonstrate extended viability (Lee et al. 2007). These models, however, also feature media perfusion or other advanced culture methods, confounding the true functional effect on hepatocytes. Elucidating the essential elements responsible for maintenance of primary phenotype is critical to the development of a simple yet effective liver model.

#### Spatial confinement leads to long-term maintenance of primary hepatocyte phenotypes

**Fig. 4:**
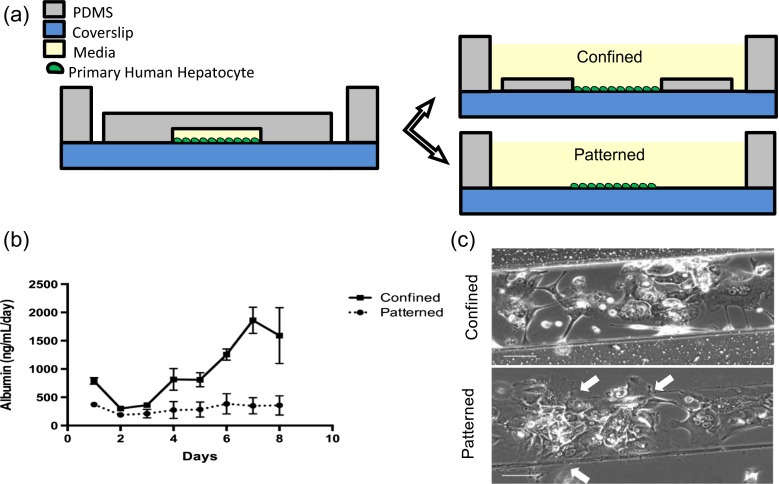
Spatial confinement is necessary to maintain hepatocyte phenotype. (**a** Devices with decreasingly complex architecture were seeded with FHH’s, and then modified post-seed to yield either a patterned strip of cells (“patterned”) or cells confined in an open channel within a well (“confined”). (**b**) Confined hepatocytes achieve stable albumin expression, but patterned hepatocytes produce much less albumin. Error bars represent SEM (n = 3). (**c** Hepatocytes within the walled device are spatially confined to the channel, but line-patterned hepatocytes are able to spread beyond their initial seeding pattern (white arrow). Scale bars represent 100 μm

The MBD possesses two key attributes that differ from well plates, which may be critical for hepatocyte culture: the microphysical environment provide by the microfluidic channel (250 μm wide, 100 μm tall), and the small volume of media available to the cells within the channel. To investigate if one or both of these differences contribute to maintenance of hepatic phenotypes, partial devices were constructed with decreasing complexity. Cells were seeded in a simple channel architecture, which was subsequently modified to yield either an open channel with walls but no top (“Confined”) or a patterned line of cells with no walls (“Patterned”) (Fig. 4a). A disparity in albumin and factor IX production resulted when hepatocytes were seeded into a confined space defined by walls, compared to hepatocytes seeded into a patterned line without confinement (Fig. 4b). Both device-like cultures featured 500 μL of media, thus the small volumes of the MBD do not seem to be sufficient to induce this effect. Microscopy revealed that hepatocytes within the patterned line were able to spread beyond their initial seeding pattern (despite the fact that the ECM coating was confined to the patterned area by the removable channel), in contrast to their wall-confined counterparts (Fig. 4c). This demonstrates the primary hepatocyte phenotype is dependent on physical confinement of the cells.

Cell concentration and compaction is a well-documented prerequisite to prevent flattening of primary hepatocytes in short-term well-based culture (LeCluyse 2001). Likewise, forming tight cords of hepatocytes within a perfused microchannel and patterning hepatocyte islands with surrounding fibroblasts has been shown to maintain proper phenotypes for long-term culture (Bhatia et al. 1998). Compaction allows for proper spacing of hepatocytes to mimic the cellular structure of a liver acinus. To supply the liver, the portal vein and hepatic artery branch 8-10 times to feed hexagonal structures called Kiernan lobules. Inside lobules, blood flows from portal venules and hepatic arterioles to the central vein of a lobule through sinusoids as plasma is filtered by sinusoidal endothelial cells before being engulfed by hepatocytes. Within the lobules are single layers of hepatocytes, often one cell thick. Fifty percent of the hepatocytes cell surface is shared with other hepatocytes. Tight junctions coordinated from cell to cell form bile canaliculi, which aggregate into a bile ductile (Arias et al. 2009). By culturing hepatocytes in a microenvironment that prevents them from spreading, and instead encouraging formation of units several cells across and 50-100 cells long, it is possible the MBD is effectively re-creating the spatial features of a liver acinus. While the exact mechanism of action of the MBD requires further investigation, this demonstrates that the spatial constriction of the channel alone, without perfusion, co-cultures, elaborate extracellular matrices or media components, is sufficient to prevent hepatocyte spreading. This understanding, especially its simplicity, is invaluable in the design of both micro-scale liver models, as well as larger liver-assist type devices.

It is important to note that the cell density was kept constant between the different conditions, to eliminate the role of cell-aggregation as a contributing factor to the phenotype. The cell density in well cultures and different device architectures was kept constant at 150,000 cells/cm^2^. Moreover, cells seeded in the MBD spread out and adhered well to the collagen-coated glass substrate, and only began to form aggregates a few days after seeding, as result of active remodeling by the cells (Fig. 2e). Though this remodeling resulted in a discontinuous layer of cells that do not appear closely packed, their cuboidal morphology appears key to their phenotype, consistent with previous data (Evenou et al. 2010). It is possible that high enough cell densities can lead to a sustained phenotype (Dembélé et al. 2014). In our hands, higher cell densities up to 300,000 cells/cm^2^ phenotypes did not improve cell phenotype in well cultures (Fig. S4). While much higher densities can maintain stable phenotypes, as in the case of spheroids (Kikuchi et al. 2009; Lee et al. 2004; Miranda et al. 2009; Thoma et al. 2005; Evenou et al. 2010), such systems can be difficult to produce, image and handle, and pose practical challenges for multiplexing. Our MBD offers a substitute for well-based attached hepatocyte culture and is simple to implement and analyze.

### HLSEC-hepatocyte co-culture under static and perfused media

As there are at least seven distinct cell types in liver (hepatocytes, cholangiocytes, endothelial cells, Kupffer cells, lymphocytes, dendritic cells, and stellate cells) and hepatocytes alone are not able to complete all the functions of the liver (Arias et al. 2009), the ability to co-culture multiple liver cells types is important to achieve a more complete liver model that reproduces many of the essential functions of the liver. To demonstrate the ability of the device to support multiple cell types, we employed the bilayer architecture to investigate juxtaposing the hepatocytes with human liver sinusoidal endothelial cells (HLSEC’s). Endothelial cells have been shown to support both hepatocyte cell line and primary hepatocyte phenotypes (Ohno et al. 2008; Lee et al. 2004). The addition of endothelial cells would also aid in developing a perfused system, as they would (along with the membrane) serve as a barrier that discourages flow from crossing from the top to the bottom channel, protecting the hepatocytes from excessive shear. To develop this vascularized device, we used the HC-04 hepatoma cell line, as cell lines are an efficient cell source for prototyping (Materne et al. 2013). Donor-specific lots of HLSEC’s were confirmed for primary phenotypes via characterization for LDL uptake and Tumor Necrosis Factor alpha-induced expression of ICAM and E-selectin. HLSEC culture was optimized to include shaking the starter flask of HLSEC’s overnight in an orbital shaker, having the effect of aligning the cells to shear stress before being seeded into the upper channel (Fig. S5).

**Fig. 5:**
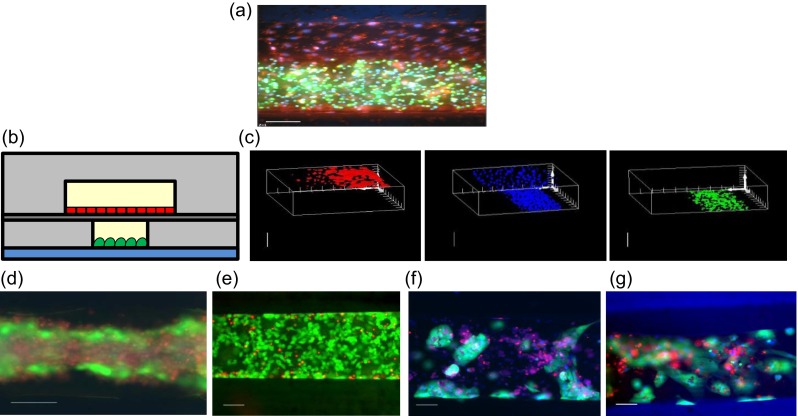
Device co-culture and perfusion. (**a**) Merged Z-stack through the co-culture device, green: HC-04, red: HLSEC, blue: DNA. (**b**) Schematic of (**a**) showing HLSEC’s in the upper channel co-cultured with HC-04 hepatocytes in the lower channel. (**c**) Three-dimensional rendering of (**a**). (**d**) HC-04 cultured in static media in the lower chamber for three weeks show overgrowth and death, but many live cells in the periphery of the cell mass in the channel. (**e**) HC-04 cultured in the lower channel under media perfusion at 2.5 μL/h for one week. HLSEC were seeded in the top channel at the start of the experiment (not imaged). (**f**) FHH’s cultured in the lower channel for three weeks under static media. (**g**) FHH’s after 12 days under static media followed by 10 days under perfusion at 137 μL/h. (**d**–**g**) Green: live, red: dead, blue: DNA. All bars represent 100 μm

While HC-04 cultured by themselves did not survive under perfusion for more than 3 days, the presence of vascular channels increased the ability of HC-04 to tolerate flow (Fig. 5). This inivestigation allow us to us to establish three basic requirements for HC-04 survival under flow, which were extrapolated for FHH. First, hepatocytes were perfused with media harvested from a culture flask, such that necessary cytokines or other extracellular factors can be maintained at appropriate concentration during perfusion. Second, establishing a monolayer of HLSEC’s in the upper, perfused channel prior to hepatocyte seeding contributed to culture longevity under flow. It is worth noting that post-experiment staining revealed no HLSEC’s remaining in the upper channel. The endothelial layer likely served to protect the hepatocytes from shear stress, gradually pulling off from the membrane while hepatocytes simultaneously were adapting to steadily increasing shear (Fig. 5b). This points to the challenges associated with culturing microvascular endothelial cells (which are generally less robust than other endothelial cells) in microfluidic channels, particularly on porous PDMS membranes. More studies are required to elucidate proper conditions for culturing these cells under shear. Third, we established the upper limit for flow rates that the hepatocytes can tolerate. This was exceptionally low in the case of HC-04 (2.5 μL/h, equivalent to a shear stress of 0.003 dyn/cm^2^ at the cell surface), indicating a loss of shear tolerance, likely due to the HC-04 cell line being adapted to static conditions over many passages. FHH’s were more tolerant, and were able to be perfused at 137 μL/h (equivalent to a shear stress of 0.1 dyn/cm^2^ at the cells surface), provided they were first cultured under static conditions to allow them enough time to attach and remodel their microenvironment prior to experiencing shear from perfusion.

A liver culture model featuring media perfusion is inherently more phenotypic because the liver is not a static organ. Previous research has shown the importance of flow for both the parenchymal and non-parenchymal aspects of the liver culture model. Healthy hepatocytes require a relatively large influx of oxygen difficult to achieve by diffusion alone, highlighting the importance of perfused oxygen-rich media (Domansky et al. 2010). Additionally, primary liver sinusoidal endothelial cells lose important phenotypes and features, such as sieve plates, after a few days in culture (Daneker et al. 1998). Efforts to maintain endothelial structure and phenotypes *in vitro* have focused on identifying proper perfusion, extracellular matrix, and cytokine gradients (Kim et al. 2013, Sato and Ohashi 2005). While our MBD is capable of perfused cell culture, much more optimization is needed to obtain *in vivo*-like vascular structures within the MBD. We have estimated the oxygen flux through the MBD to be greater than that in a typical wellplate culture, negating the need for flow to meet oxygen demands. Moreover, static culture is advantageous because there is no need for syringe pumps and tubing. The MBD’s compatibility with different cell types and flow conditions is important for assessing which biological and mechanical components are necessary for a fully optimized liver model.

## Conclusion

Here we present a simple and flexible liver model capable of supporting human hepatocytes and their phenotypes for at least three weeks of continuous culture. When hepatocytes are cultured in the MBD alone in static media, spatial confinement provided by the channel walls promotes albumin production, factor IX production, expression of cytochrome P450 enzymes, and formation of functional bile canaliculi, indicating that hepatocytes are maintaining functionality and demonstrating the potential of this liver model. As organ-on-a-chip models are often complex and tailored for specific cell types or organ models, this device demonstrates flexibility in that either hepatic cell lines or primary hepatocytes can be seeded into the parenchymal chamber, and if needed, additional cell types such as sinusoidal endothelial cells, stellate cells, or Kupffer cells can be utilized for co-culture effects and as potential cues for improved long-term liver culture. Furthermore, the MBD can be perfused or kept static, depending on the ideal culture conditions for each cell type within the MBD. Further studies with different media perfusion rates, supporting cell types, and complex extracellular matrices can elucidate and measure the incremental improvement each has on *in vitro* liver models. These findings are essential to development of a long-term, phenotypic, reproducible and multiplexed liver disease model

## Electronic supplementary material

Below is the link to the electronic supplementary material.

Fig. S1 Scanning electron microscopy images of the PDMS membrane, taken from a top-down view (a) and at a 40° tilt (b-c). Images show the holes to be fully open (PDF 249 kb)

Fig. S2 Device optimization for long term hepatocyte culture. (a) Early renditions of the MBD featured channels 15 mm in length, leading to a dead zone (red: dead cells, green:, live cells) in the middle 5 mm of the channel when cells were cultured under static conditions. To decrease diffusion distances from the middle of the channel to channel-ending media reservoirs, devices were shortened such that channels are 7 mm long, making static culture possible throughout the channel. (b) Three different membrane materials were tested for device construction and biocompatibility: (left to right) PDMS, polyethelyne terephthalate (PET) and polycarbonate (PC). PET and PC membrane devices were assembled with glue and periodically delaminated during use as noted by cells leaving the channel (white arrows). Also, PDMS membranes diffract less light than PET and PC membranes, leading to more clear phase contract imaging. (c) Primary hepatocytes cultured within PDMS membrane devices demonstrate stable albumin expression, whereas PET membrane devices do not. Errors bars represent SEM (n = 4) (PDF 358 kb)

Fig. S3 Flow modeling within bilayer devices. (a) A prototype device with a cross-sectional-area ratio (cross sectional area of the top channel divided by cross-sectional area of the bottom channel) of 2 was modeled for how flow is affected by the membrane. Modeling in COMSOL predicted a slight cross-flow through the membrane and down the lower channel during upper channel perfusion. Tests using dye demonstrated this phenomenon. (b) Modeling predicted that transfer between channels to be strongly affected by cross-sectional-area ratios. Thus, the MBD used in this study was designed with a cross-sectional-area ratio of 4 (PDF 255 kb)

Fig. S4 Albumin production as a function of cell density. FHH’s were cultured in well plates at different densities, ranging from 37,500 to 300,000 cells/cm2. In all cases, albumin production declined over the course of 8 days (PDF 217 kb)

Fig. S5 Characterization and optimization of HLSEC. (a) HLSEC take up labeled low-density lipoprotein (top, white), this phenotype is absent in a fibroblast control (bottom). (b) HLSEC exposed to TNFα (top) or DMSO control (bottom) show increased expression of E-selectin (green, blue; DNA). (c) Fewer HLSEC’s show expression of ICAM-1 without TNFα (bottom), but nearly 100 % show expression after TNFα exposure (top, green: ICAM1, blue: DNA). (d) HLSEC without shaking (left) and after orbital shaking at 1 000 rpm overnight (right); directional shear from orbital shaking aligns cells to condition them for perfused microfluidic culture. Scale bars represent 100 μm (PDF 282 kb)
