# Supplementary material for: Microphysical space of a liver sinusoid device enables simplified long-term maintenance of chimeric mouse-expanded human hepatocytes
Source: Biomed Microdevices. 2014 Jun 7;16(5):727–36. doi: 10.1007/s10544-014-9877-x (PMC4152623; doi:10.1007/s10544-014-9877-x)
Supplement: Supplementary file 1 — Scanning electron microscopy images of the PDMS membrane, taken from a top-down view (a) and at a 40° tilt (b-c). Images show the holes to be fully open (PDF 249 kb) [file 10544_2014_9877_MOESM1_ESM.pdf]

Title

Microphysical space of a liver sinusoid device enables simplified long-term maintenance of chimeric mouse-expanded human hepatocytes

Journal

Biomedical Microdevices

Authors

Steven P. Maher[1,2], Richard B. Crouse[1], Amy J. Conway[1], Emilee C. Bannister[1], Anil Kumar H. Achyuta[1], Amy Y. Clark[1], Francy L. Sinatra[1], Joseph D. Cuiffi[1], John H. Adams[2], Dennis E. Kyle*[2] and Wajeeh M. Saadi*[1]
[1]Bioengineering Center at USF, Charles Stark Draper Laboratory, 3802 Spectrum Blvd ste 201, Tampa, Florida 33612; telephone (813) 465-5488, fax: (813) 465-5401; wsaadi@draper.com
[2]Department of Global Health, University of South Florida, 3720 Spectrum Blvd ste 304, Tampa, Florida 33612; telephone: (813) 974-1273, fax: (813) 974-0992; dkyle@health.usf.edu
*corresponding authors

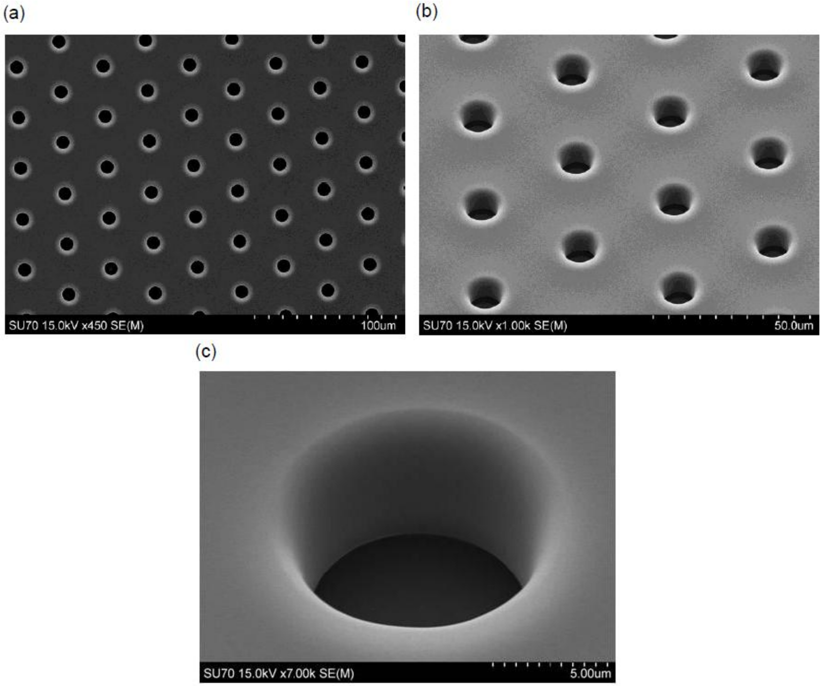

S1 Scanning electron microscopy images of the PDMS membrane, taken from a top-down view (a) and 40° tilt (b-c). Images show the holes to be fully open.
