# Supplementary material for: Microphysical space of a liver sinusoid device enables simplified long-term maintenance of chimeric mouse-expanded human hepatocytes
Source: Biomed Microdevices. 2014 Jun 7;16(5):727–36. doi: 10.1007/s10544-014-9877-x (PMC4152623; doi:10.1007/s10544-014-9877-x)
Supplement: Supplementary file 2 — Device optimization for long term hepatocyte culture. (a) Early renditions of the MBD featured channels 15 mm in length, leading to a dead zone (red: dead cells, green:, live cells) in the middle 5 mm of the channel when cells were cultured under static conditions. To decrease diffusion distances from the middle of the channel to channel-ending media reservoirs, devices were shortened such that channels are 7 mm long, making static culture possible throughout the channel. (b) Three different membrane materials were tested for device construction and biocompatibility: (left to right) PDMS, polyethelyne terephthalate (PET) and polycarbonate (PC). PET and PC membrane devices were assembled with glue and periodically delaminated during use as noted by cells leaving the channel (white arrows). Also, PDMS membranes diffract less light than PET and PC membranes, leading to more clear phase contract imaging. (c) Primary hepatocytes cultured within PDMS membrane devices demonstrate stable albumin expression, whereas PET membrane devices do not. Errors bars represent SEM (n = 4) (PDF 358 kb) [file 10544_2014_9877_MOESM2_ESM.pdf]

Title

Microphysical space of a liver sinusoid device enables simplified long-term maintenance of chimeric mouse-expanded human hepatocytes

Journal

Biomedical Microdevices

Authors

Steven P. Maher[1,2], Richard B. Crouse[1], Amy J. Conway[1], Emilee C. Bannister[1], Anil Kumar H. Achyuta[1], Amy Y. Clark[1], Francy L. Sinatra[1], Joseph D. Cuiffi[1], John H. Adams[2], Dennis E. Kyle*[2] and Wajeeh M. Saadi*[1]

[1]Bioengineering Center at USF, Charles Stark Draper Laboratory, 3802 Spectrum Blvd ste 201, Tampa, Florida 33612; telephone (813) 465-5488, fax: (813) 465-5401; wsaadi@draper.com

[2]Department of Global Health, University of South Florida, 3720 Spectrum Blvd ste 304, Tampa, Florida 33612; telephone: (813) 974-1273, fax: (813) 974-0992; dkyle@health.usf.edu

*corresponding authors

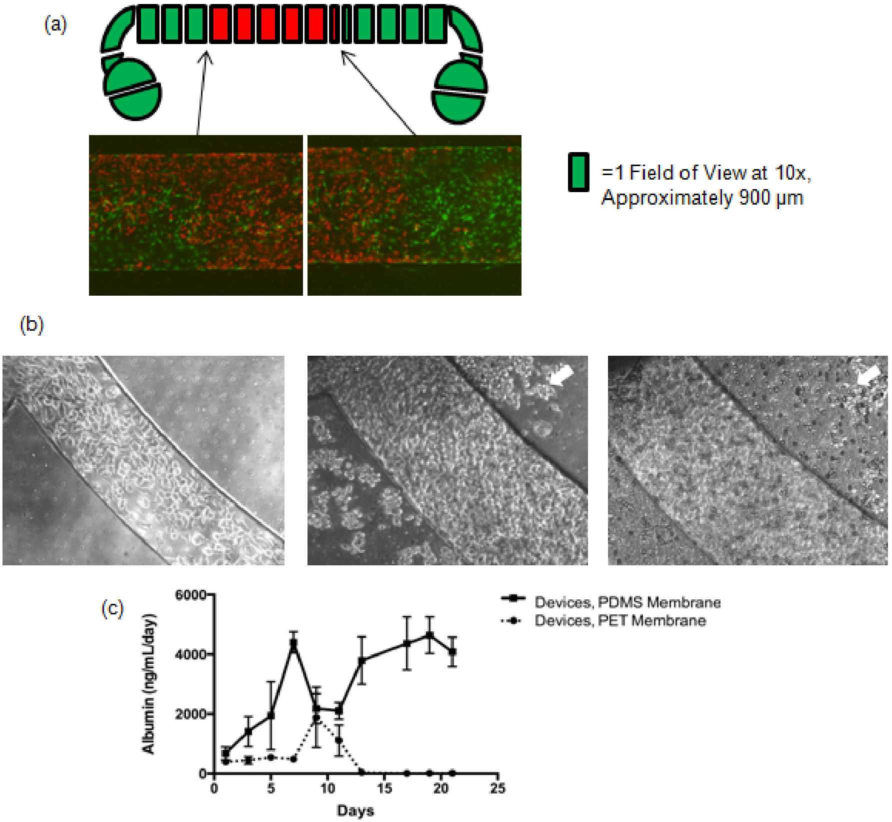

Fig. S2 Device optimization for long term hepatocyte culture. (a) Early renditions of the MBD featured channels 15 mm in length, leading to a dead zone (red: dead cells, green:, live cells) in the middle 5 mm of the channel when cells were cultured under static conditions. To decrease diffusion distances from the middle of the channel to channel-ending media reservoirs, devices were shortened such that channels are 7 mm long, making static culture possible throughout the channel. (b) Three different membrane materials were tested for device construction and biocompatibility: (l to r) PDMS, PET and PC. PET and PC membrane devices were assembled with glue and periodically delaminated during use as noted by cells leaving the channel (white arrows). Also, PDMS membranes diffract less light than PET and PC membranes, leading to more clear phase contract imaging. (c) Primary hepatocytes cultured within PDMS membrane devices demonstrate stable albumin expression, whereas PET membrane devices do not. Errors bars represent SEM (n=4).
