# Supplementary material for: Microphysical space of a liver sinusoid device enables simplified long-term maintenance of chimeric mouse-expanded human hepatocytes
Source: Biomed Microdevices. 2014 Jun 7;16(5):727–36. doi: 10.1007/s10544-014-9877-x (PMC4152623; doi:10.1007/s10544-014-9877-x)
Supplement: Supplementary file 3 — Flow modeling within bilayer devices. (a) A prototype device with a cross-sectional-area ratio (cross sectional area of the top channel divided by cross-sectional area of the bottom channel) of 2 was modeled for how flow is affected by the membrane. Modeling in COMSOL predicted a slight cross-flow through the membrane and down the lower channel during upper channel perfusion. Tests using dye demonstrated this phenomenon. (b) Modeling predicted that transfer between channels to be strongly affected by cross-sectional-area ratios. Thus, the MBD used in this study was designed with a cross-sectional-area ratio of 4 (PDF 255 kb) [file 10544_2014_9877_MOESM3_ESM.pdf]

Title

Microphysical space of a liver sinusoid device enables simplified long-term maintenance of chimeric mouse-expanded human hepatocytes

Journal

Biomedical Microdevices

Authors

Steven P. Maher[1,2], Richard B. Crouse[1], Amy J. Conway[1], Emilee C. Bannister[1], Anil Kumar H. Achyuta[1], Amy Y. Clark[1], Francy L. Sinatra[1], Joseph D. Cuiffi[1], John H. Adams[2], Dennis E. Kyle*[2] and Wajeeh M. Saadi*[1]
[1]Bioengineering Center at USF, Charles Stark Draper Laboratory, 3802 Spectrum Blvd ste 201, Tampa, Florida 33612; telephone (813) 465-5488, fax: (813) 465-5401; wsaadi@draper.com
[2]Department of Global Health, University of South Florida, 3720 Spectrum Blvd ste 304, Tampa, Florida 33612; telephone: (813) 974-1273, fax: (813) 974-0992; dkyle@health.usf.edu
*corresponding authors

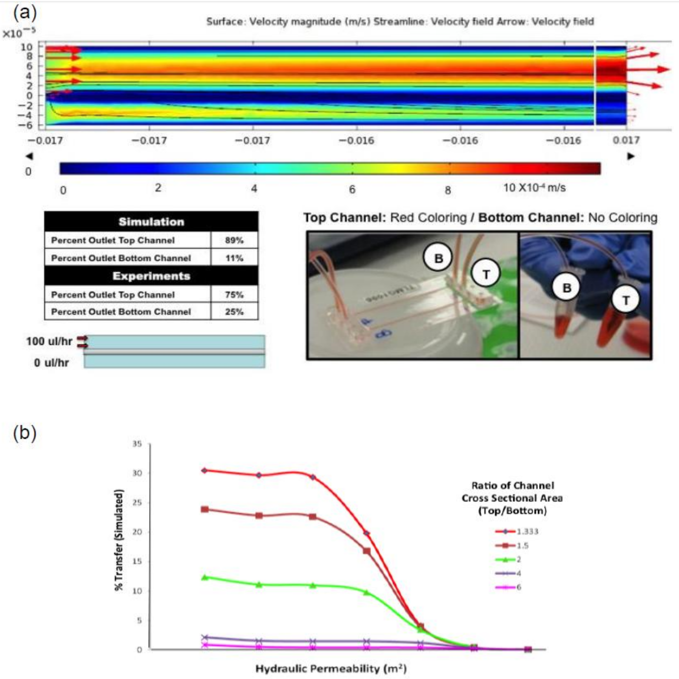

Fig. S3 Flow modeling within bilayer devices. (a) A prototype device with a cross-sectional-area ratio (cross sectional area of the top channel divided by cross-sectional area of the bottom channel) of 2 was modeled for how flow is affected by the membrane. Modeling in COMSOL predicted a slight cross-flow through the membrane and down the lower channel during upper channel perfusion. Tests using dye demonstrated this phenomenon. (b) Modeling predicted that transfer between channels to be strongly affected by cross-sectional-area ratios. Thus, the MBD used in this study was designed with a cross-sectional-area ratio of 4
