# Supplementary material for: Microphysical space of a liver sinusoid device enables simplified long-term maintenance of chimeric mouse-expanded human hepatocytes
Source: Biomed Microdevices. 2014 Jun 7;16(5):727–36. doi: 10.1007/s10544-014-9877-x (PMC4152623; doi:10.1007/s10544-014-9877-x)
Supplement: Supplementary file 4 — Albumin production as a function of cell density. FHH’s were cultured in well plates at different densities, ranging from 37,500 to 300,000 cells/cm2. In all cases, albumin production declined over the course of 8 days (PDF 217 kb) [file 10544_2014_9877_MOESM4_ESM.pdf]

Title

Microphysical space of a liver sinusoid device enables simplified long-term maintenance of chimeric mouse-expanded human hepatocytes

Journal

Biomedical Microdevices

Authors

Steven P. Maher[1,2], Richard B. Crouse[1], Amy J. Conway[1], Emilee C. Bannister[1], Anil Kumar H. Achyuta[1], Amy Y. Clark[1], Francy L. Sinatra[1], Joseph D. Cuiffi[1], John H. Adams[2], Dennis E. Kyle*[2] and Wajeeh M. Saadi*[1]
[1]Bioengineering Center at USF, Charles Stark Draper Laboratory, 3802 Spectrum Blvd ste 201, Tampa, Florida 33612; telephone (813) 465-5488, fax: (813) 465-5401; wsaadi@draper.com
[2]Department of Global Health, University of South Florida, 3720 Spectrum Blvd ste 304, Tampa, Florida 33612; telephone: (813) 974-1273, fax: (813) 974-0992; dkyle@health.usf.edu
*corresponding authors

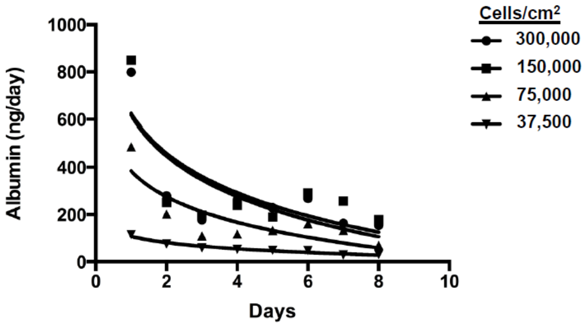

Fig. S4 Albumin production as a function of cell density. FHH's were cultured in well plates at different densities, ranging from 37,500 to 300,000 cells/cm2. In all cases, albumin production declined over the course of 8 days.
